# Lady Strangford Hospital, Port Said

**Published:** 1891-08-22

**Authors:** 


### Lady Strangford Hospital, Port Said.

The Rev. John Scarth, Hon. Sec. of the above hospital, writing from Bearsted Vicarage, Maidstone, says: The enlargement of the Lady Strangford Hospital is now urgently needed; in the month of June all the beds were full; a ward for infectious cases, separate from the rest of the building is specially desired. The Committee would venture to appeal for donations, and with your kind permission this letter may draw attention to the subject.

To be landed at Port Said because too ill to continue the voyage, whether that voyage be to England, to India, or to Australia, must surely be a misfortune hard to bear. Happily, however, there is now this good hospital ready to receive any such sufferers. More than 1,100 have been received within the last four years.

The traffic through the Canal increases, and the average of cases for this year is equal to nearly one additional patient per day; in the first six months there were 177.

The hospital has, as yet, no capital to draw from; indeed, the Committee are still answerable for part of the first cost. The annual contributions, fortunately, meet the expenses, and the great value of a well-managed English hospital, with an English doctor and English nursing sisters as the staff, has been proved but cannot be estimated. Of course the majority of the patients are sailors, but a few passengers, and some of the residents also, have been received.

His Royal Highness the Duke of Edinburgh, K.G., has kindly sent £10. The Right Hon Lord Brassey, K.C.B., has sent £100; Mrs. Jowers, £50; Mr. W. Sturdy, £10 10s.; Mr. George Gunings, £10; Right Hon. Sir E. Malet, £5; the Lady Ermyntrude Malet, £5; Sir John Coode, K.C.M.G., £5; the Board of Trade, £100; the Trinity House, £10; some leading shipowners, about £200.

It will be remembered that a British hospital at Port Said was originally selected as the national memorial to General Gordon, and when nearly £20,000 had been subscribed the plan was changed and, instead of the hospital at Port Said, a Boys' Home in England was adopted and nearly all the sum transferred. The need for the hospital was still as great, but to collect money a second time for the same object made the task all the more difficult. Nevertheless, the hospital was provided; and just when the late Viscountess Strangford was on her way out to inaugurate the work in which she had taken so much interest, she was called to her rest and reward; therefore the hospital has henceforth borne her name, and the work has been blessed. It will probably become more and more important.

Contributors will please cross their cheques "Imperial Ottoman Bank," and make them payable to Lieutenant-General Sir John Stokes, K.C.B., the Treasurer, Good Rest, Haywards Heath.

Mr. H. R. Greene writes from Southsea: Readers of the Rev. Mr. Scarth's appeal on behalf of the Lady Strangford Hospital at Port Said might suppose that, till the deserving institution whose claims he advocates was established, there existed no place at Port Said where sick seamen or passengers landed from passing vessels could be received and properly treated. Such, however, is far from being the case; for many years the Egyptian Government has maintained a very well-appointed hospital in Port Said, where many thousands of sick or infirm wayfarers have received as good treatment and care as they could in any "well-managed English hospital." There is room in Port Said for both establishments, but it is only fair that credit should be fairly distributed.

